# Statistical analysis plan for the Prophylactic Melatonin for Delirium in Intensive Care (ProMEDIC): a randomised controlled trial

**DOI:** 10.1186/s13063-020-04981-0

**Published:** 2021-01-05

**Authors:** Bradley Wibrow, F. Eduardo Martinez, Andrew Ford, Erin Kelty, Kevin Murray, Kwok M. Ho, Edward Litton, Erina Myers, Matthew Anstey

**Affiliations:** 1 Intensive Care Unit, Sir Charles Gairdner Hospital, University of WA, Perth, WA Australia; 2 Intensive Care Unit, John Hunter Hospital, University of Newcastle, Newcastle, NSW Australia; 3 Dept. of Psychiatry, Royal Perth Hospital, Perth, WA Australia; 4 Centre for Applied Statistics, University of WA, Perth, WA Australia; 5 Intensive Care Unit, Royal Perth Hospital, Medical School, University of Western Australia & Murdoch University, Perth, Australia; 6 St. John of God Hospital Subiaco, Intensive Care Unit, Fiona Stanley Hospital, Perth, WA Australia; 7 Intensive Care Unit, Sir Charles Gairdner Hospital, Perth, WA Australia; 8 Intensive Care Unit, Sir Charles Gairdner Hospital, Curtin University, Perth, WA Australia

**Keywords:** Delirium, Melatonin, Intensive care unit, Sleep

## Abstract

**Rationale:**

Delirium is defined as acute organic brain dysfunction characterised by inattention and disturbance of cognition. It is common in the intensive care unit and is associated with poorer outcomes. Good quality sleep is important in the prevention and management of delirium. Melatonin is a natural hormone secreted by the pineal gland which helps in the regulation of the sleep-wake cycle. It is possible that melatonin supplementation in intensive care improves sleep and prevents delirium.

**Methods and design:**

The ‘Prophylactic Melatonin for Delirium in Intensive Care’ study is a multi-centre, randomised, double-blinded, placebo-controlled trial. The primary objective of this study is to determine whether melatonin given prophylactically decreases delirium in critically ill patients. A total of 850 ICU patients have been randomised (1:1) to receive either melatonin or a placebo. Participants were monitored twice daily for symptoms of delirium.

**Results:**

This paper and the attached additional files describe the statistical analysis plan (SAP) for the trial. The SAP has been developed and submitted for publication before the database has been locked and before the treatment allocation has been unblinded. The SAP contains details of analyses to be undertaken, which will be reported in the primary and secondary publications.

**Discussion:**

The SAP details the analyses that will be done to avoid bias coming from knowledge of the results in advance. This trial will determine whether prophylactic melatonin administered to intensive care unit patients helps decrease the rate and the severity of delirium.

**Trial registration:**

Australian and New Zealand Clinical Trial Registry (ANZCTR) ACTRN1261600043647, registration date: 06 April 2016. WHO Trial Number – U1111-1175-1814

## Introduction

Delirium is a condition of acute organic brain dysfunction with disturbances of attention and cognition, a fluctuating course, and occurring as a direct consequence of an underlying medical condition [1]. It is particularly common in patients in intensive care units (ICU), with a wide range of prevalence reported in the literature (11–80%) [2–4]. Delirium in ICU is associated with poorer outcomes, including higher mortality, more time on a ventilator, increased length of stay (LOS), greater post-operative complications, increased need for tracheostomy, increased medical costs, and higher risk of dementia and cognitive impairment [3, 5–12].

Good quality sleep at night is recognised as an important aspect in the prevention and management of delirium. Melatonin is a natural hormone secreted by the pineal gland, which helps in the regulation of the sleep-wake cycle. Decreased melatonin levels and fluctuations in its metabolite, 6-SMT, have been associated with delirium in post-surgical and intensive care patients [13, 14].

It is possible that supplementing melatonin improves sleep and prevents delirium. A number of clinical trials of melatonin or melatonin agonists for the prevention of delirium have been performed to date [15–26]. It is difficult to draw firm conclusions due to conflicting results, methodological issues, and patient heterogeneity.

The ‘Prophylactic Melatonin for Delirium in Intensive Care’ is a multi-centre randomised double-blinded, placebo-controlled trial. The primary objective of this study is to determine whether melatonin given prophylactically decreases delirium in critically ill patients.

This study may provide evidence for a simple and effective agent to reduce delirium in patients admitted to the ICU.

## Study methods

This is a multi-centre, randomised, double-blinded, placebo-controlled trial of melatonin versus placebo to decrease the rate of delirium in ICU. A total of 850 ICU patients have been randomised on a 1:1 ratio to receive either melatonin or a placebo. Study participants were administered an oral dose of melatonin 4 mg enterally or placebo once daily at 21:00hs. for up to 14 days or until discharge from the ICU. Participants were monitored twice daily for symptoms of delirium. Additionally, data is being collected on the duration and severity of delirium, sleep quality, hospital and ICU length of stay (LOS), duration of mechanical ventilation, and mortality.

Patients were randomised according to a computer-generated randomisation list, stratified by site, with standard block sizes of 6. Variable block sizes were considered unlikely to provide additional benefit, as the study drug and placebo have the same appearance and all clinical staff are blinded, thus maintaining allocation concealment. The randomisation list has been maintained by the University of Western Australia Centre for Applied Statistics.

The patients, investigators, ICU doctors, and nurses looking after patients enrolled in the study are blinded to the treatment that the patients are receiving. Only the University of Western Australia Centre for Applied Statistics, the compounding pharmacy, and the independent Data Safety Monitoring Committee (DSMC) (should they need to, in order to investigate an adverse event) have had access to the treatment allocation list.

Based on the sample size calculations, with an alpha value of 0.05 and a power of 80%, a total of 850 patients have been recruited to the study, with 425 patients in each arm. Estimates of sample size were made based on the percentage of delirium-free assessments (the primary outcome) and, secondarily, the ICU LOS. LOS was chosen in addition to the percentage of delirium-free assessments as a significant relationship between delirium and LOS has been observed. Delirium assessment is by its nature subjective and the investigators wished to also allow the study to be powered to detect a reduction of LOS of 1 day (as an objective patient-orientated outcome). An audit of 100 patients across the two primary sites was used to provide preliminary data to guide sample size calculations where an average of 54% of assessments were deemed delirium free (standard deviation of ± 45%). The research group is interested in being able to detect a 10% increase in the percentage of delirium-free assessments. This would require a sample size calculation of 319 per group. Adjustment for non-parametric data (15%) increases this to 367 per group. It is expected that up to 10% of patients recruited in the study will not be assessable for delirium at any point during the study. To account for this, and missing data/loss to follow-up, the sample size was increased by 15% to 423 patients per group (total rounded to 850).

To detect a change in LOS of 1 day, using a sample size calculation for a gamma distribution, 387 patients per group would be required. The greater number of 850 was selected to have enough power to answer both questions. The formula used to calculate sample size was that described by Wand and Chow in 2007 [27].

The framework of the study is of superiority of melatonin over placebo for the prevention of delirium. Superiority analyses will be two-sided and considered statistically significant at the 5% level.

The DSMC is comprised of an independent intensivist, an epidemiologist, and a statistician independent of the trial statistician. The independent DSMC will review all serious adverse events and perform an interim analysis after 400 patients using the Haybittle-Peto stopping rule. The Haybittle-Peto stopping rule states that if an interim analysis shows a probability of equal to or less than 0.001 that a difference as extreme or more between the treatments is found, given that the null hypothesis is true, then the trial should be stopped early [28].

Statistical analysis of data will all occur collectively once all data, including follow-up at 90 days, has been collected. Outcome assessments were undertaken during ICU stay, at discharge from ICU, at discharge from hospital, and at 28 and 90 days.

## Statistical principles

Differences will be considered statistically significant when *P* values are < 0.05, and 95% confidence intervals (95% CI) will be reported.

Adherence to protocol will be considered when patients have received all the required doses while in ICU and all the CAM-ICU assessments. This will be presented with descriptive statistics as a percentage and numerator and denominator. Protocol deviation is defined as lack of compliance with study drug administration or lack of assessment with CAM-ICU. All protocol deviations will be collated and presented in the results as descriptive statistics. All analyses will be conducted on both an intention-to-treat (ITT) and per-protocol (PP) basis. The ITT sample is defined as all randomised subjects including those who are lost to follow-up or have missing data. If the patient does not receive the assigned intervention post-randomisation, the patient will still be included in the ITT sample and analysed according to assigned treatment group.

## Trial population

Screening data will be presented as descriptive statistics. Patients eligible to be considered for the study must be admitted to the ICU with an expected total LOS of at least 72 h, as determined by the treating intensivist. Eligible participants also must be able to be enrolled and treated within 48 h of being admitted to the ICU.

Exclusion criteria for participation in the study includes (1) patients aged < 18 years; (2) patients already on melatonin before their admission to ICU; (3) prior hypersensitivity reaction to any of the components of the study drug (melatonin, sucralose and glycerol, marshmallow flavour); (4) patients expected to be discharged within 72 h of their ICU admission; (5) expected/inevitable death within next 48 h; (6) pregnant or breastfeeding; (7) non-English speaking; (8) patients that are not expected to improve adequately to be able to be assessed with a CAM-ICU score during their ICU stay; (9) patients that are not able to be assessed due to neurological problems that would affect their ability to participate in a CAM-ICU assessment, as judged by treating physician; (10) no enteral route, as melatonin is not available in intravenous formulation; and (11) hepatic impairment defined as alanine transferase (ALT) > 500 IU/L, previous liver transplant, or liver cirrhosis categories Childs-Pugh B and C.

**Fig. 1:**
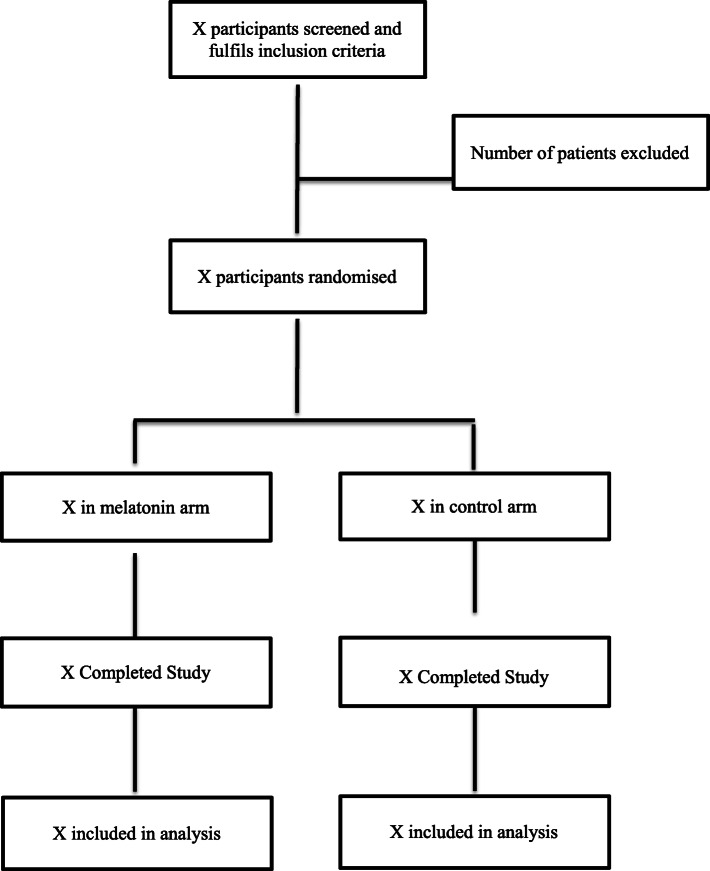
Statistical analysis plan flowchart version 1

A CONSORT flow diagram will be presented. (Fig. 1) This will include the number of patients assessed for eligibility, excluded, randomised, allocated to each intervention arm, followed up, and analysed for each group. A report of withdrawals will be provided, describing the level at which they have occurred, the timing of withdrawals, and the reasons for the withdrawals.

Baseline characteristics including age, gender, severity scores, source of admission, and diagnosis will be presented as a table and summarised.

## Analysis

Primary and secondary outcomes will be analysed as described below. Where appropriate, analysis of primary and secondary outcomes will be analysed by a number of predetermined subgroups. These subgroups will include (1) percentage of delirium-free assessments by age: less than 65 or more than 65 years of age; (2) percentage of delirium-free assessments by gender; (3) percentage of delirium-free assessments by delirium subgroup at baseline: hyperactive, hypoactive, and mixed, as well as no delirium; (4) percentage of delirium-free assessments by diagnostic category (respiratory, cardiovascular, gastrointestinal, toxicological, neurological, metabolic, sepsis, trauma, traumatic brain injury); and (5) percentage of delirium-free assessments by > 50% risk or < 50% risk as per Pre-Deliric Score (calculated from first 24 h of ICU admission). Multiplicity adjustments will not be made.

For the primary outcome of percentage of delirium-free episodes, patients will be assessed for delirium twice a day for 14 days or until they are discharged from ICU, whichever occurs first. The CAM-ICU tool will be used to assess delirium, with a result recorded as either ‘positive’ or ‘negative’ for delirium. The assessment will be carried out by trained ICU medical or nursing staff. The percentage of delirium-free assessments will be calculated for each participant. The percentage of delirium-free assessments for participants in each group will then be compared using Student’s *t* test. It is assumed that the data will be normally distributed. If the distribution of the data is not normally distributed, alternative approaches will be considered; however, the initial plan is robust to deviations from normality with this sample size. Supporting analysis will use linear regression making adjustments for baseline covariates.

A secondary analysis will look at the rates of delirium in each group. The number of times an individual is reported as being positive for delirium and the total number of assessments will be examined by a comparison of rates. In addition, a Poisson regression will be used with the number of positive delirium assessments reported and offset the total number of assessments. Baseline characteristics will be included as covariates in the model. The covariates will include age, gender, delirium subtype, diagnostic category, and Pre-Deliric score.

Finally, an analysis that looks at whether the individual reported a positive delirium assessment during their stay will be undertaken by comparing proportions between the groups through a logistic regression analysis controlling for variables including age, gender and length of stay.

The secondary outcomes to be analysed include (1) severity and duration of delirium, (2) sleep quality, (3) ICU and hospital length of stay;,(4) duration of mechanical ventilation, (5) mortality, and (6) economic analysis. Each secondary outcome will be adjusted for the same covariates as mentioned before: age, gender, delirium subtype, diagnostic category, and Pre-Deliric score.

### Severity and duration of delirium

All analyses, with the exception of ‘Delirium and Coma Free Days’, will use either a linear mixed model approach or generalised linear mixed model approach. Both of these will include a random individual effect to account for the repeated nature of the data and an appropriate correlation structure. Estimated differences between the groups and 95% confidence intervals will be provided throughout. The following analyses will be undertaken:

1. CAM-S: Score: CAM-S scores range from 0 (no delirium) to 7 (severe delirium). Linear mixed models will be used to compare CAM-S score as a response between the two treatment groups for individuals who have tested positive using CAM-ICU.
2. Delirium and Coma-Free Days: Poisson regression will be used to compare the count of delirium and coma-free days between the treatment groups using an offset of number of days in ICU.
3. Anti-psychotics or sedation: The use of anti-psychotics or sedations will be recorded as a binary outcome labelled ‘used’ or ‘not used’ for each day during the study period. Generalised linear mixed models with logit link will be used to compare the two treatment groups with respect to the binary outcome.
4. Physical restraint: Each day during the study period, the use of physical restraints will be recorded as a binary outcome labelled having been ‘used’ or ‘not used’. Generalised linear mixed models with logit link will be used to compare the use of restraints between the two treatment groups with respect to the binary outcome.
5. Participation with physiotherapy and mobilisation: Ability of participate in physiotherapy and mobilisation sessions will be assessed as a binary outcome labelled ‘yes’ or ‘no’. Generalised linear mixed models with logit link will be used to compare the use of the two treatment groups with respect to the binary outcome.
6. Patient removal of lines: Each day during the study period, it will be recorded as whether or not the patient has attempted to or successfully removed their lines (binary outcome labelled ‘yes’ or ‘no’). Generalised linear mixed models with logit link will be used to compare the used to compare the use of the two treatment groups with respect to the binary outcome.
7. Type of delirium: At each assessment, patients with delirium will be classified as having either hyperactive, hypoactive, mixed delirium, or no delirium. Generalised linear mixed models for ordinal response will be used to compare the use of the two treatment groups with respect to the ordinal outcome.

### Sleep quality

Sleep quality will be assessed through sleep questionnaires, estimation of hours of sleep, and polysomnography.

1. Sleep questionnaires: Two types of sleep questionnaire will be utilised during the study. The first, the RCSQ provides a score out of 50. The second (Little’s) involved a mixture of dichotomous questions and five-point rating questions [29, 30].
2. Estimated hours of sleep: For both questionnaire responses, calculated outcomes will be compared between the treatment groups using Student’s *t* tests and supporting linear regression models to adjust for covariates.
3. Polysomnography: In the subset of patients with polysomnography, the following will be analysed: total sleep time, sleep efficiency, percentage REM sleep, arousal index, and odds ratio product.

All calculated outcomes will be compared between the treatment groups using Student’s *t* tests and supporting linear regression models to adjust for covariates.

### ICU and hospital length of stay

Initial comparisons between the two groups for ICU and hospital length of stay will be compared using Student’s *t* test. Supporting analysis will also be carried out using Poisson regression, accounting for previously described covariates. Length of stay will be analysed in particular with the aforementioned subgroups.

### Duration of mechanical ventilation

The total number of hours of mechanical ventilation will be calculated for each participant. Comparison between the two groups will be made using Student’s *t* test. Supporting analysis will also be carried out using Poisson regression, accounting for covariates such as age, gender, admission type and diagnosis type.

### Mortality

Mortality (time to death) will be analysed using Kaplan-Meier curves and Cox proportional hazard models, both unadjusted and adjusting for previously described covariates. Individuals who did not experience an event (death) will be censored at the end of the follow-up period of the study. Individuals withdrawn from the study early or lost to follow-up will be censored at the date of withdrawal or the last contact visit respectively. Results will be presented using hazard ratios and 95% confidence intervals for relative comparisons between the groups and absolute risk and differences will be estimated at both 28 and 90 days.

### Costing analysis

The cost of ICU care has been estimated at $4375 [15] and $5534 (Medical Division SCGH, 2015) per day based on national and state data respectively. From these figures, any reduction in costs through reduced LOS will be calculated. The cost per melatonin dose ($20 AUD per 30 ml bottle of 2 mg/ml) as well as consumables (syringe for NGT application) will also be calculated. There would also be a small ‘time’ cost associated with administration, but we believe it could reasonably be included in the general nursing workload. Costs will be presented in Australian dollars, adjusted to 2020.

### Missing data

Due to the nature and implementation of the CAM-ICU tool, it is expected there will be some missing data. However, such data is expected to be missing at random and consequently appropriate analyses will be carried out. The patterns of data availability for primary and secondary outcomes and reasons for missingness, where known, will be summarised for the two treatment groups. In situations where mixed modelling is the primary analysis, missing data will be handled by the likelihood-based estimation used in these analyses where the MAR assumption holds. In other situations of missing data, analyses will be carried out, if applicable, using multiple imputation.

It is expected that the source of missing data will be predominantly the dependent variable (based on missed delirium free assessments and outcomes). Consequently, we will not use multiple imputation techniques if this is the case and simply report the missingness patterns. However, if missing data is present in other variables, we will use these techniques, utilising the multiple imputation with chained equations (MICE) methodology [31].

### Harms

Safety will be evaluated by tabulation of adverse events and will be presented with descriptive statistics at baseline and follow-up visits for each treatment group. With each SAE, the study investigator will determine the intensity and causality as per ICH GCP guidelines All SAEs will be discussed at the Data Safety Monitoring Committee meetings to reach a consensus on causality. If this differs from the original decision the local ethics committee is informed.

The number and proportion of patients experiencing at least one AE, and the number and proportion of patients experiencing at least one SAE will be presented descriptively. The mean number of AEs and SAEs per patient will be presented. In addition, the frequencies of patients with (i) adverse events and (ii) serious adverse events will both be compared between the intervention groups using Fisher’s exact tests or chi-squared tests.

Data analysis will be carried out using the Stata MP, version 15. This SAP has been written in accordance with published guidelines for the content of statistical analysis plans in clinical trials [27].

## Trial status

At the time of publication, the ProMEDIC study had completed enrolment and follow-up of outcome data was ongoing. The statistical analysis plan of the study has been submitted for publication to this peer-reviewed journal before completion of follow-up and unblinding.

## Data Availability

There is no data from which conclusions have been made to accompany this manuscript.

## Abbreviations

CAM-ICU: Confusion assessment method for ICU
CAM-S: Confusion assessment method-severity
DSMC: Data safety monitoring committee
ICU: Intensive care unit
ITT: Intention to treat
LOS: Length of stay
PP: Per protocol
SAP: Statistical analysis plan
